# STRATEGIES AND OUTCOMES OF GAIT SPEED IMPLEMENTATION IN A GERIATRIC PRIMARY CARE CLINIC

**DOI:** 10.1093/geroni/igae098.1914

**Published:** 2024-12-31

**Authors:** Alexander Garbin, Thomas Johnson, Caitlin Allison, Hillary Lum

**Affiliations:** VA Eastern Colorado Healthcare System, Aurora, Colorado, United States; University of Colorado School of Medicine, Aurora, Colorado, United States; University of Colorado Health, Aurora, Colorado, United States; University of Colorado School of Medicine, Aurora, Colorado, United States

## Abstract

Over a 10-month period, gait speed was measured during 4700 patient visits (1931 unique patients) as part of a quality improvement project within a geriatric primary care clinic. This presentation will comprehensively detail strategies and outcomes from this implementation project. Primary implementation strategies included use of an automated gait speed measurement device to reduce Medical Assistant burden, integration of gait speed into the electronic medical record, identification of clinical champions, and development of environmental supports to facilitate gait speed measurement and provider referral guidance. Burden was assessed at the start of the implementation period (n=124 visits), with results detailing a median of 19.5 seconds (IQR 14.3, 26.4) added to rooming workflow with the addition of gait speed measurements. Implementation outcomes of reach and adoption were examined on a weekly basis and were defined by the proportion of patient visits with a gait speed measurement and proportion of provider notes that included gait speed, respectively. At the end of the implementation period, reach was 73.3% while adoption was 53.9%. We also examined gait speed change in patients with two or more gait speed measurements, separated by at least 180 days (n=516). After stratifying by baseline risk (<0.6 m/s ‘at risk’; ≥0.6m/s ‘not at risk’), patients identified as ‘at risk’ (n=159) increased their gait speed by 0.07 m/s (IQR -0.01, 0.20; p<0.001), while those ‘not at risk’ (n=419) experienced no change (IQR -0.12, 0.11; p=0.94). Together, this information details considerations for implementing gait speed while also demonstrating its preliminary feasibility and benefit.

